# Residential Area Sociodemographic and Breast Cancer Screening Venue Location Built Environmental Features Associated with Women’s Use of Closest Venue in Greater Sydney, Australia

**DOI:** 10.3390/ijerph182111277

**Published:** 2021-10-27

**Authors:** Jahidur Rahman Khan, Suzanne J. Carroll, Neil T. Coffee, Matthew Warner-Smith, David Roder, Mark Daniel

**Affiliations:** 1 Australian Geospatial Health Laboratory, Health Research Institute, University of Canberra, Canberra, ACT 2617, Australia; Suzanne.Carroll@canberra.edu.au (S.J.C.); neil.coffee@canberra.edu.au (N.T.C.); mark.daniel@canberra.edu.au (M.D.); 2 School of Health Sciences, University of South Australia, Adelaide, SA 5000, Australia; david.roder@unisa.edu.au; 3 Housing and Healthy Cities Research Group, School of Architecture and Built Environment, University of Adelaide, Adelaide, SA 5005, Australia; 4 Cancer Institute NSW, St Leonards, Sydney, NSW 2065, Australia; Matthew.WarnerSmith@health.nsw.gov.au; 5 Department of Medicine, St. Vincent’s Hospital, The University of Melbourne, Fitzroy, VIC 3065, Australia

**Keywords:** breast screening, closest screening venue attendance, locational features, observational study, multilevel modelling

## Abstract

Understanding environmental predictors of women’s use of closest breast screening venue versus other site(s) may assist optimal venue placement. This study assessed relationships between residential-area sociodemographic measures, venue location features, and women’s use of closest versus other venues. Data of 320,672 Greater Sydney screening attendees were spatially joined to residential state suburbs (SSCs) (*n* = 799). SSC-level sociodemographic measures included proportions of: women speaking English at home; university-educated; full-time employed; and dwellings with motor-vehicles. A geographic information system identified each woman’s closest venue to home, and venue co-location with bus-stop, train-station, hospital, general practitioner, and shop(s). Multilevel logistic models estimated associations between environmental measures and closest venue attendance. Attendance at closest venue was 59.4%. Closest venue attendance was positively associated with SSC-level women speaking English but inversely associated with SSC-level women university-educated, full-time employed, and dwellings with motor-vehicles. Mobile venue co-location with general practitioner and shop was positively, but co-location with bus-stop and hospital was inversely associated with attendance. Attendance was positively associated with fixed venue co-location with train-station and hospital but inversely associated with venue co-location with bus-stop, general practitioner, and shop. Program planners should consider these features when optimising service locations to enhance utilisation. Some counterintuitive results necessitate additional investigation.

## 1. Introduction

Preventive screening programs target the early detection of conditions, including breast cancer, to achieve early treatment and reduce mortality [1]. As screening is voluntary, it is important to optimise the placement or siting of screening venue locations to ensure these programs are convenient not only in terms of their geographic accessibility, but also available public transit and options for shopping or errand running, and accessing health care services [2]. Facility locations and accessibility of the facilities are acknowledged as key factors influencing the success of preventive healthcare programs [1,3], including breast cancer screening (BCS).

Breast cancer is the most frequently diagnosed cancer among women worldwide and a leading cause of mortality [4]. It is also the most prevalent cancer among women in Australia, where early breast cancer detection is available via a national BCS program called *BreastScreen Australia* [5]. This organised population-based program provides free mammography on a biennial basis to asymptomatic women aged 40 years and older [6]. The program targets women aged 50–74 years through invitation letters, whereas women aged 40–49 and >74 years can access screening services if they choose but do not receive biennial invitations. The program aims to screen at least 70% of women aged 50–74 years biennially [6]. National BCS uptake rates in Australia remain below this target, with 54.3% of women aged 50–74 years screened in 2015–2016 [5].

Research indicates that greater distance to screening venue (geographical accessibility) is inversely related to BCS participation [7,8,9,10]. However, distance is not the only factor to determine participation [1]. One study suggested that some women may find it more convenient to attend a screening venue near their workplace than the venue closest to home [11]. Immigrant women may prefer a screening venue with radiographers speaking their language, irrespective of venue location. For example, in Toronto, Canada, Chinese immigrants preferred Chinese-speaking family physicians regardless of location [12].

Features of women’s residential areas and screening venue locations’ built environment may also relate to venue utilisation. Residential area sociodemographic features refer to the sociodemographic composition of the resident population as expressed at residential area spatial boundaries. Built environmental features of the venue location refer to the human-made physical features of the surroundings of the location where the screening venue is located. Women living in urban regions, higher-income neighbourhoods, and areas with longer average travel time to their workplaces are unlikely to use their closest BCS venue [13]. It has been suggested that women living in an area served by multiple BCS venues may screen at a venue other than their closest where the other venue is co-located with a shopping centre, thus supporting other tasks performed in tandem with a screening appointment [1]. Other considerations, including accessibility by public transport and non-clinical ambience, may also be relevant to the screening venue used [14,15,16]. Understanding residential area and venue location features associated with screening at one’s closest versus other venue is important as BCS programs seek to optimise the placement of screening venues not only in terms of accessibility for their target populations but also the features that will attract and support women’s attendance. Few studies have assessed the relationship between residential area sociodemographic factors, venue location features and the choice of closest versus other (i.e., more distal) BCS venue.

Whilst Australian research has assessed the influence of distance to screening venue on women’s BCS participation [9,10], no Australian study published thus far has investigated area-level and venue location factors associated with women screening at their closest versus other screening venue location. Such research has a strong potential to inform decisions for program planning to optimise the placement of BCS venues to support BCS uptake.

The aims of this study were twofold: (1) to assess the associations between residential area sociodemographic features and women’s use of their closest versus other BCS venue; and (2) to assess the associations between closest venue location features and women’s use of their closest venue, and differences in these associations for mobile and fixed venues.

## 2. Materials and Methods

### Population, Setting and Breast Cancer Screening Data

This observational study was undertaken for the Greater Sydney region of NSW, Australia. Greater Sydney comprised one-fifth of the Australian population in 2011 [17] and had lower BCS participation (49.5% among women aged 50–69 years in 2015–2016) than the national rate (54.6%) [5,18].

Population-based data were provided by the Cancer Institute of New South Wales (CINSW), who are responsible for the NSW *BreastScreen Australia* program. The CINSW provided residential address geocoded data for 323,914 women residing in Greater Sydney who participated in screening between January 2011 and December 2014. Residential geocodes enabled spatial joins of individual-level data with state suburbs (SSC) and related Census-based sociodemographic data (Australian Bureau of Statistics [ABS] 2011). An SSC is a census-specific area formed by aggregating Statistical Area Level 1 (SA1s), where an SA1 is the smallest geographic unit at which 2011 census data are available [19]. SSC is a close approximation of a gazetted suburb. SSCs were used as they are sufficiently large to examine population features but small enough to identify spatial concentrations of social disadvantage [20]. The CINSW also provided geocoded screening venue location data enabling the calculation of distances between residences and venues.

Records were excluded for women who live in Greater Sydney but screened at a venue outside of Greater Sydney (*n* = 1490) and whose screening date corresponded to a holiday (e.g., Christmas, New Year’s) (*n* = 994). Women missing data for language spoken at home, or for whom distance to venue could not be calculated (due to, e.g., living on islands, or not near the road network), or who resided in SSCs with less than ten screeners and/or less than ten women aged ≥15 years and/or less than ten dwellings (*n* = 758) were excluded. This yielded an analytic dataset of 320,672 records within 799 SSCs.

## 3. Measures

### 3.1. Outcome Measure: Use of Closest Breast Cancer Screening Venue (Individual Level)

Road-network distances between women’s geocoded residences and screening venues were calculated using MapInfo Pro (V16), RouteFinder (V5) and StreetPro, 2011 (Pitney Bowes, New York, NY, USA). Women were then classified as having screened at the venue closest to their home or at a more distal venue based on the rank order of distances.

### 3.2. Predictors

#### 3.2.1. Area-Level

Predictors of screening at the closest venue were residential SSC sociodemographic measures: proportions of women speaking English at home, university-educated (Bachelor’s Degree or higher), and full-time employed; and proportion of dwellings with motor vehicle ownership. These data were extracted from the ABS 2011 Census data [19] for the SSC in which each screener resided.

#### 3.2.2. Venue Location

Venue location features included: type of screening facility (fixed or mobile); and co-location with bus-stop, train-station, hospital, general practitioner, and shop(s). Screening venues (*n* = 71) were either “fixed” (i.e., a permanent screening facility, *n* = 21) or “mobile” (i.e., a location where a mobile screening van temporarily provides service for a discrete period, *n* = 50).

Venue location geocodes enabled the characterisation of venues as co-located with a bus-stop, train-station, hospital, general practitioner, or shop(s). Co-location was defined as the presence of a built environmental feature within 500 m (800 m for a train station) of a BCS venue [21]. The different distance for bus stops and train stations correspond to public transport catchment distances representing standard walking distances for transit users [22]. The 500/800 m road-network buffers were created centred on each venue location using ArcGIS Desktop 10.5 Network Analyst extension (generalised option) and 2011 NSW road network data (source: StreetPro, 2011, Pitney Bowes) [21].

Public transport data were extracted from General Transit Feed Specification transit data (3 November 2013) [23]. Venue co-location with a bus stop (or train station) was determined as the availability of at least one bus stop (or train station) within the relevant 500 m (or 800 m) road-network buffer [21].

Data for hospitals, general practitioners, and shops were extracted from the 2011 MapInfo Business Points Australia dataset based on 2006 Australian and New Zealand Standard Industrial Classification codes. These service and commercial features were coded as mutually exclusive with a priority order of hospital, general practitioner, and shop, given that multiple combinations of these services could be co-located [21]. This study chose mutually exclusive features because we were interested in evaluating the unique impact (i.e., statistical independence) of a hospital, general practitioner, or shop on screening participation. The order itself was based on the reality that screening venues are often based at hospitals, and general practitioners and shops are also often co-located with hospitals. An example would be general practitioners providing services within the hospital, and gift shops within the hospital. In such a situation it was hypothesised that the impact of co-location with the hospital would be more important than the impact of general practitioners and gift shops contained within the hospital.

#### 3.2.3. Covariates

Covariates within this study included individual-level information on woman’s age at screening (years) and whether English was the primary language spoken at home (yes/no).

### 3.3. Statistical Analysis

Descriptive statistics were calculated for outcome and exposure measures. Hot spot analysis to identify spatial clustering of women’s use of closest venue was carried out using the Getis-Ord Gi* statistic [24] (using the Hot Spot Analysis tool in ArcGIS Pro V2.6.2 [25], ESRI, Redlands, CA, USA). To identify clusters of high or low values across space, this statistic evaluated the value at a given location in relation to values at neighbouring locations (distance band of 1000 m). The Gi* statistic returned Z score for each location, with Z scores greater than 1.96 (or less than −1.96) indicating statistically significant hot spots (or cold spots), and Z scores near to zero indicating no significant clusters at a significance level of 5%.

In this study, individuals were nested within residential areas (i.e., SSCs). A suitable analytical technique for such nested data is the multilevel model, allowing simultaneous estimation of the effects of area- and individual- level factors on individual-level outcome (i.e., closest venue attendance) while accounting for area clustering. Ignoring the area-level clustering can lead to biased standard errors of model parameter estimates [26]. Multilevel logistic regression models were used to estimate the associations between SSC-level sociodemographic measures, closest venue location features and women’s use of closest venue. A two-level model was used with individual women (level 1) nested within SSCs (level 2). This model allows quantifying the joint contribution of the individual- and SSC-level factors on women’s use of closest venue. Intra-class correlations (ICCs) [27] and median odds ratios (MORs) [28] were calculated in the multilevel logistic model to quantify unexplained variation in closest venue attendance rates across SSCs.

To assess Aim 1, five separate models were constructed. Individual-level covariates were included in all models. Models were first fitted for each sociodemographic variable, and then all sociodemographic variables were modelled together. Prior to analysis, SSC-level sociodemographic measures were standardised (mean = 0, standard deviation [SD] = 1) to enable comparison of their relative effects. To assess Aim 2, five separate sets of models were constructed for both the closest fixed and mobile venue location features, with individual-level covariates and area-level sociodemographic measures included in all models. This stratified analysis for mobile and fixed venues was done to check whether there is any difference in direction association between venue location features and women’s use of closest venue by venue type. Data analyses were conducted using R version 3.6.1 statistical software [29] (R Foundation for Statistical Computing, Vienna, Austria).

## 4. Results

Table 1 presents the characteristics of screened women, SSCs and venue locations included in the analytic dataset. Fifty-nine percent of women attended their closest venue, the average age was sixty years, and most women spoke English at home (68.7%). For SSCs, the mean proportion of women speaking English at home was similar, aligning with the individual-level data. The proportion of dwellings reporting motor vehicle ownership was 88%. The mean proportion of women with university education was 23% and 27% for women employed full time. Most venues were mobile (*n* = 50), 77.5% of venues were co-located with bus stops, 39.4% train stations, 16.9% hospitals, 54.9% general practitioners, and 22.5% with shops. 

**Figure 1:**
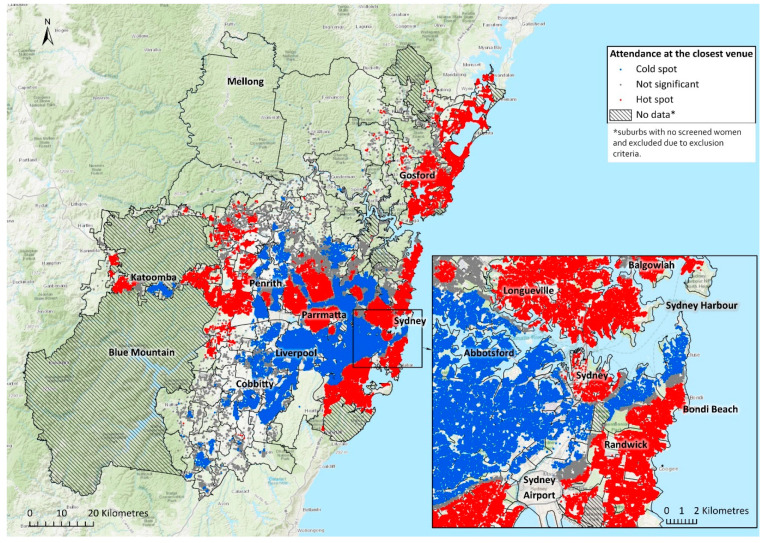
Hot and cold spots of the use of closest breast screening venue in Greater Sydney with central Sydney area as inset.

Spatial analysis identified the distribution of statistically significant “hot spots” (greater densities of women attending their closest venue) and “cold spots” (lesser densities of women attending their closest venue) in Greater Sydney (Figure 1). Most hotspots were in the northern and central SSCs, followed by some western and west-central SSCs. Cold spots were located primarily in southwestern SSCs, followed by western and southern SSCs.

Residential area sociodemographic features associated with women’s use of their closest venue are shown in Table 2. In models featuring a single area-level sociodemographic measure, a greater likelihood of using the closest venue among screeners was predicted by residing in an SSC with a greater proportion of women speaking English at home (AOR 1.529; 95% CI 1.410 to 1.659), whilst a lesser likelihood of using the closest venue was predicted by residing in an SSC with greater proportions of university-educated women (AOR 0.901, 95% CI 0.826 to 0.982), and full-time employed women (AOR 0.834, 95% CI 0.765 to 0.909). However, SSC-level proportion of dwellings with a motor vehicle was not associated with screeners’ use of closest venue.

In the omnibus model including all sociodemographic features, a greater proportion of women speaking English at home (AOR 1.613, 95% CI 1.476 to 1.763), and a greater proportion of full-time employed women (AOR 0.843, 95% CI 0.763 to 0.932), remained statistically significant, and the proportion of dwellings with motor vehicles reached statistical significance (AOR 0.870, 95% CI 0.795 to 0.952). SSC-level proportion of university-educated women was not associated with screeners’ use of their closest venue in the omnibus model.

Among individual-level characteristics, older women were less likely to use their closest venue than younger-aged women across all models (omnibus model, AOR 0.985, 95% CI 0.983 to 0.986). Individual-level English spoken at home was not statistically significant in any of the models.

Variance inflation factors indicated no substantial multicollinearity between predictors in the full sociodemographic model (i.e., less than 5). The full model, including all area sociodemographic measures, performed best per Akaike’s information criterion (AIC = 350,561.391). The ICC (28.22%) and MOR (2.96) of this model indicated substantial heterogeneity in screeners’ use of closest venue between SSCs.

Table 3 presents the estimated associations between features of the closest screening venue and women’s use of their closest venue for models stratified by closest venue type (i.e., mobile or fixed site). The likelihood of closet venue attendance was greater when the closest mobile venue was co-located with a general practitioner (AOR 1.066, 95% CI 1.008 to 1.126) and a shop (AOR 1.071, 95% CI 1.010 to 1.135), but was lesser when the closest mobile venue was co-located with a bus stop (AOR 0.574, 95% CI 0.535 to 0.616) and a hospital (AOR 0.889, 95% CI 0.808 to 0.978). There was no significant association between closest mobile venue co-location with train-station and venue attendance. Screeners were more likely to use the closest fixed venue when co-located with train-station (AOR 1.868, 95% CI 1.785 to 1.955) and hospital (AOR 1.123, 95% CI 1.086 to 1.161). In contrast, the likelihood of attending the closest fixed venue was lesser when co-located with a bus stop (AOR 0.862, 95% CI 0.824 to 0.903), general practitioner (AOR 0.899, 95% 0.869 to 0.929), and a shop (AOR 0.454, 95% CI 0.322 to 0.641).

All models were adjusted for individual level measures: age at screening, language spoken at home, and SSC level measures (standardised): proportion of women who speak only English at home; proportion of women with at least Bachelor’s degree; proportion of full time employed women; proportion of dwellings with at least one motor vehicle.

## 5. Discussion

This is the first study to assess residential area sociodemographic features and venue location features in assessing predictors of women screening at their geographically closest venue in Greater Sydney. Fifty-nine percent of women who screened for breast cancer during January 2011–December 2014 in Greater Sydney attended the venue closest to their residence. Substantial spatial clustering of women using a venue other than their closest (i.e., cold spots) supports the relevance of tailored programmatic initiatives delivered to certain geographic areas to improve local screening rates as well as strategies for optimising the placement of venues. It is important, therefore, to understand which local area features predict women screening at their closest venue. Thus far, only one study (in the USA) has reported on residential area sociodemographic factors in relation to women’s use of their closest breast screening venue [13]. No previous Australian study has assessed this question, and thus the current study represents an important first step in exploring these associations in the Australian context.

This study indicates that residential area sociodemographic features predict screening venue attendance. Women residing in areas with greater proportions of women speaking English at home were more likely to use the venue closest to their residence. No studies have previously assessed this influence. Research on cultural diversity has reported, however, that culturally diverse women may prefer to use health services with matching language-specific capabilities. A study on the utilisation of family physicians in Toronto reported that Chinese immigrants prefer Chinese-speaking family physicians [12]. This suggests that women of non-English speaking backgrounds may attend a more distant venue known to support their language preference. Our result can also be viewed through the lens of cultural connectivity. Women may receive information regarding screening services and venue locations from culturally similar others in their residential area, influencing venue attendance. For instance, Arabic-speaking migrants tended to get their health information from their own cultural group (friends, family, community members) rather than from health services [30].

In contrast, women residing in areas with greater proportions of university-educated (single area predictor model only) and full-time employed women were more likely to use a venue other than the closest. Education supports access to health information and empowerment in health-related decision making [31,32]. Education could support other venue use through access to information about screening sites beyond the closest and planning appointments and attendance based on other factors (e.g., work-related constraints). That the association between area education and venue attendance attenuated to the null on inclusion of other sociodemographic measures is unsurprising given the correlations between these measures, particularly area employment. Employed women may prefer to use a screening venue near their workplace rather than their place of residence [11] due to convenience and the opportunity to screen pre- or post-work with only limited impact on work attendance. This study did not have any information on individual-level reasons for using a venue other than the closest.

Our study also found that the use of a distal venue was greater among women residing in an area characterised by greater access to motor vehicles. Motor vehicle ownership or availability likely reduces transport-related barriers to accessing more distal venues. A study from the UK suggested that affluent patients who own cars could be more willing to travel further to see a preferred healthcare provider [33].

Overall, our findings on residential-area sociodemographic features suggest placing screening venues near areas with high proportions of English-speaking women may support local venue utilisation. Conversely, placing venues near areas with high proportions of non-English speaking women may require the provision of additional factors (e.g., culturally diverse staff, interpreter services, linguistically and culturally relevant educational materials) to optimise venue utilisation. Our previous study focused on screening uptake found a lesser uptake in areas with a greater density of non-English speaking at home women [21], supporting the need for attention to these areas. For areas with high rates of full-time employed women, extended opening hours may assist local screening attendance. Women who work full-time may also benefit from having a venue close to their workplace.

Of the venue location built environmental features assessed in this study, most of the features were associated with venue attendance; however, the patterns of associations varied according to venue type (i.e., mobile or fixed). Women were more likely to use venues *other* than the closest, regardless of venue type, if the venue was co-located with bus stops, an intuitively comprehensible result. On the other hand, the odds of using the closest fixed venue was greater when co-located with a train-station. On the face of it, this is a non-intuitively comprehensible result given the relevance of train transport to attending a local or at least the closest screening venue. It is possible however that for some women the closest venue might indeed be most efficiently reached by train, avoiding heavy Sydney traffic and parking problems. There was no association between venue attendance and *mobile* venue co-location with train stations. Bus routes tend to take longer to travel to destinations due to circuitous routes. Train lines are more direct, may have the capacity to transport more people and take less time than a bus from the same location. These factors may, to some degree, explain the varying effects of public transit stops on venue attendance, but further research is needed. Regardless, these findings support locating fixed screening sites near train stations.

Co-location with hospital, general practitioner, and shop were also associated with venue attendance. For fixed venues, screeners were more likely to attend the closest venue where co-located with a hospital but more likely to use other venues than the closest if it were a mobile venue co-located with a hospital. This supports provision of fixed services at or near hospitals but not the placement of mobile screening sites near hospitals. Notably, there were only four mobile sites co-located with hospitals in Greater Sydney during our study period and each of these sites would have provided services only for a short time span (e.g., 5 weeks [34]). In contrast, there were 8 fixed venues providing screening services throughout the entire study period. The infrequent co-location of mobile venues with hospitals and their limited time availability during the study period may have impacted our findings; women had less opportunity to access services provided by a mobile van co-located with a hospital than a fixed service at a hospital.

Screening attendance at closest venues was more likely for mobile venues co-located with general practitioners or shops; conversely, screening at other than the closest was more likely when the closest venue was a fixed venue co-located with general practitioners and shops. General practitioners may promote mobile venue visits to women in advance of actual visits and encourage attendance by educating patients regarding the importance of screening. Screening promotion by general practitioners may be less when they are co-located with a fixed screening site. For co-location with shops, a mobile van parked/co-located with a shopping centre will likely be placed to maximise visibility [35], thus publicising breast screening and current availability of the service, whereas a fixed site within a shopping centre may be less effective in publicising its presence. Our findings support the placement of mobile services near general practitioners and shops.

Aside from these environmental factors, this study assessed two individual-level factors, age and speaking English at home, in relation to venue attendance. Individual-level speaking English as the primary language at home was not related to venue attendance. Regarding age, older women were more likely to attend a venue other than their closest, which could be due to venue preference based on previous interaction with the system (e.g., venue loyalty [36]).

This study has several key strengths. It addresses an important research gap by assessing residential area sociodemographic features and venue location built environmental predictors of breast screening venue attendance in an Australian context making use of large population-based screening participation and spatial datasets. The large administrative screening participation data used in this study represent the population of women screened through *BreastScreen Australia*. These population data avoid any sampling bias. These data are reliable, avoid social desirability, reporting and recall bias that are common concerns with self-reported data, and their use allows for population-level inference. Other strengths include using objective, rigorous distance measures (i.e., the road-network distance between residence and venue locations) rather than perceived or Euclidean distance measures to determine venue attendance, and the conduct of spatial and multilevel analyses. Multilevel analysis is advantageous because it enables the estimation of associations between both area and individual level factors with closest venue attendance as well as estimating area clustering. The percentage of total variation in nearest screening venue attendance attributable to between-area differences was estimated using area clustering. Importantly, failure to account for area-level clustering when modelling nested data may result in biased standard errors of model parameter estimates [26]. In terms of limitations, this study was able to account for only limited individual-level sociodemographic data. Other individual-level data (e.g., education, employment, car ownership, activity space, use of transport modes, preference, and general practitioner recommendation) and venue information (e.g., the number of machines and staff, after-hours or weekend screening services) not available for inclusion in this study could conceivably impact venue attendance. Furthermore, some women (e.g., those of high socioeconomic status [37]) may choose to attend a private screening service rather than the *BreastScreen Australia* program. Private screening service utilisation data were not available in this investigation. Women’s use of private screening may impact estimations relating to screening attendance versus non-attendance but are less likely to impact on the findings in this study as the current analyses are focused only on women who have screened, particularly those who screened using the *BreastScreen Australia* service. This may reduce the generalisability of the findings reported here to women who use a private screening service. Lastly, the estimated relationships between area-level sociodemographic features and individuals’ venue attendance as investigated in this study may not support an analogous inference about the relationships between individual-level sociodemographic features and individuals’ venue attendance.

## 6. Conclusions

In Greater Sydney, just under three out of five women who screened for breast cancer attended their closest screening venue. Residential area sociodemographic measures and venue co-location factors were associated with venue attendance. The provision of services near areas with high proportions of linguistically diverse women may require additional culturally appropriate supports to encourage local venue attendance. Services near areas with high rates of full-time employed people may consider extending their opening hours. Findings also support locating fixed screening venues near train stations and hospitals and mobile venues near general practitioners and shops. These findings can be used to improve service delivery and placement of screening venues. Some counterintuitive results highlight the need for future research to improve the understanding of the role of locational features in closest venue attendance.

## Figures and Tables

**Table 1:** Descriptive statistics for the analytic dataset and distribution of environmental features.

Measure	Statistics		
**Individual level characteristics** (*n* = 320,672)	**Mean (SD)**		
Attended closest venue for screening (%)	59.4		
Age at screening (years)	59.6 (6.4)		
Speaking English at home (yes) (%)	68.7		
**SSC-level features** (proportions; *n* = 799 SSCs)			
Women speaking English at home ^1^	70.9 (21.2)		
Women’s education ^2^	22.7 (12.1)		
Women’s employment (full-time) ^3^	27.4 (7.4)		
Motor vehicle ownership ^4^	88.1 (9.4)		
**Venue features** (*n* = 71 venues)	All venues	Fixed (*n* = 21)	Mobile (*n* = 50)
Co-located with bus stop, (%)	77.5	85.7	74.0
Co-located with train station, (%)	39.4	57.1	32.0
Co-located with hospital, (%)	16.9	38.1	8.0
Co-located with general practitioner, (%)	54.9	57.1	54.0
Co-located with shop, (%)	22.5	4.8	30.0

SD: standard deviation; ^1^ proportion of women who speak only English at home; ^2^ proportion of women with at least Bachelor’s degree; ^3^ proportion of full-time employed women; ^4^ proportion of dwellings with at least one motor vehicle.

**Table 2:** Associations between SSC-level sociodemographic features and women’s use of their closest breast screening venue (*n* = 320,672).

Model Specification	Women Speaking English at Home	Women’s Education	Women’s Employment (Full Time)	Motor Vehicle Ownership	All Sociodemographic Measures
Measure	AOR (95% CI)	AOR (95% CI)	AOR (95% CI)	AOR (95% CI)	AOR (95% CI)
**SSC-level features**					
Women speaking English at home ^1^	1.529 (1.410 to 1.659)	-	-	-	1.613 (1.476 to 1.763)
Women’s education ^2^	-	0.901 (0.826 to 0.982)	-	-	1.009 (0.912 to 1.116)
Women’s employment (full time) ^3^	-	-	0.834 (0.765 to 0.909)	-	0.843 (0.763 to 0.932)
Motor vehicle ownership ^4^	-	-	-	1.073 (0.984 to 1.170)	0.870 (0.795 to 0.952)
**Individual-level characteristics**					
Age at screening	0.985 (0.983 to 0.986)	0.985 (0.983 to 0.986)	0.985 (0.983 to 0.986)	0.985 (0.983 to 0.986)	0.985 (0.983 to 0.986)
Speaking English at home (Ref.: No)	1.000	1.000	1.000	1.000	1.000
Yes	0.987 (0.967 to 1.006)	0.991 (0.972 to 1.011)	0.991 (0.972 to 1.011)	0.991 (0.971 to 1.011)	0.986 (0.967 to 1.006)
ICC_ssc_	0.2888	0.3144	0.3113	0.3151	0.2822
MOR_ssc_	3.0119	3.2269	3.1998	3.2335	2.9590
AIC	350,580.666	350,673.232	350,661.838	350,676.320	350,561.391

AOR: adjusted odds ratio; CI: confidence interval; ICC: intra-class correlation; MOR: median odds ratio; AIC: Akaike information criteria; ^1^ proportion of women who speaking only English at home (standardised); ^2^ proportion of women with at least Bachelor’s degree (standardised); ^3^ proportion of full-time employed women (standardised); ^4^ proportion of dwellings with at least one motor vehicles (standardised).

**Table 3:** Associations between closest venue (mobile and fixed) location built environmental features and women’s use of their closest breast screening venue.

Model Specification	Co-Location with Bus Stop	Co-Location with Train Station	Co-Location with Hospital	Co-Location with General Practitioner	Co-Location with Shop
Measure	AOR (95% CI)	AOR (95% CI)	AOR (95% CI)	AOR (95% CI)	AOR (95% CI)
**Closest venue location features**					
**Mobile venue** (*n* = 71,380)					
Co-location with bus stop	0.574 (0.535 to 0.616)	-	-	-	-
Co-location with train station	-	0.964 (0.898 to 1.034)	-	-	-
Co-location with hospital	-	-	0.889 (0.808 to 0.978)	-	-
Co-location with general practitioner	-	-	-	1.066 (1.008 to 1.126)	-
Co-location with shop	-	-	-	-	1.071 (1.010 to 1.135)
ICC_ssc_	0.3185	0.3097	0.3093	0.3114	0.3086
MOR_ssc_	3.2633	3.1863	3.1827	3.2011	3.1771
AIC	76,676.530	76,916.897	76,912.060	76,912.833	76,912.639
**Fixed venue** (*n* = 249,292)					
Co-location with bus stop	0.862 (0.824 to 0.903)	-	-	-	-
Co-location with train station	-	1.868 (1.785 to 1.955)	-	-	-
Co-location with hospital	-	-	1.123 (1.086 to 1.161)	-	-
Co-location with general practitioner	-	-	-	0.899 (0.869 to 0.929)	-
Co-location with shop	-	-	-	-	0.454 (0.322 to 0.641)
ICC_ssc_	0.3262	0.3148	0.3265	0.3266	0.3231
MOR_ssc_	3.3332	3.2310	3.3354	3.3364	3.3045
AIC	261,964.382	261,246.709	261,957.882	261,964.573	261,982.366

AOR: adjusted odds ratio; CI: confidence interval; ICC: intra-class correlation; MOR: median odds ratio; AIC: Akaike information criteria.

## Data Availability

No additional data available.
